# The Relevance of the Research on the Psychosocial Dimensions of Aging Is Really the Same in Europe and USA?

**DOI:** 10.2174/1745017901915010008

**Published:** 2019-01-23

**Authors:** Federica Sancassiani, Ferdinando Romano, Antonio Preti

**Affiliations:** 1 Department of Medical Sciences and Public Health, University of Cagliari, Cagliari, Italy; 2 Department of Public Health and Infectious Diseases, University of Rome “La Sapienza”, Rome, Italy

The paper by Kirilov and Coll. [1] has the merit of focusing on a strongly critical general theme: the weight given to the results of literature in Europe and the USA. The importance of the article is still increased by the fact that it examines the specific theme of active aging, a fundamental theme, especially for Europe, given the demographic trends of the old continent [2, 3].

The fact that the subject matter is everywhere relevant, but somewhat more critical in Europe, would mean an imbalance of interest between the two countries. However, the results of the research reassure us: apparently, the projects financed with public money in Europe and in the US have the same impact in the literature.

We used the term “reassuring” as Europeans because, on the contrary, Europe, and even more after the 'Brexit', could, in some respects, appear less competitive.

As a matter of fact, the role of scientific journals is influential on the impact of publishing. If one consults the Scimago ranking of the top 20 scientific journals, when the British are left aside, there is no European journal on the list. Among the first 100 scientific journals of the Scimago ranking, 52 are from the USA, 36 from the UK and only 12 from European countries, of which 8 are Dutch (where there is a historical tradition) 3 German and 1 Swedish [4]. Moreover, some recent reports in the literature on the evaluation of scientific production for the academic career in certain countries of the European Union would suggest that the impact of scientific research is not given a proper weight [5, 6]. Overall, in the specific theme of psychosocial interventions, a brief analysis of some contextual reviews, would suggest a much greater weight of American research compared to the European one [7-10].

Indeed, the same authors pointed out that while in the US' projects it is mandatory to bring into view the source of funding on possible publications, this aspect is not equally emphasized in European projects. This, by itself, is already an indicator of less attention paid to the impact of the publications.

Despite the merits of the Kirilov *et al.*’s article, there is a limit that should be overcome by future research: the paper does not report the amount of funding. The Horizon / FP7 calls do finance projects on average larger than NIH. If there were any disparities in the funds collected, even with the same number of papers published, this would not show an equal impact between the two shores of the Atlantic. This hypothesis must be verified.
